# Prolonged ketamine infusion modulates limbic connectivity and induces sustained remission of treatment-resistant depression

**DOI:** 10.1007/s00213-021-05762-6

**Published:** 2021-01-22

**Authors:** Joshua S. Siegel, Ben J. A. Palanca, Beau M. Ances, Evan D. Kharasch, Julie A. Schweiger, Michael D. Yingling, Abraham Z. Snyder, Ginger E. Nicol, Eric J. Lenze, Nuri B. Farber

**Affiliations:** 1 Department of Psychiatry, Washington University School of Medicine, 660 S. Euclid, Box 8134, St. Louis, MO 63110 USA; 2 Department of Anesthesiology, Washington University School of Medicine, St. Louis, MO USA; 3 Department of Neurology, Washington University School of Medicine, St. Louis, MO USA; 4 Duke Anesthesiology, Durham, NC USA; 5 Department of Radiology, Washington University School of Medicine, St. Louis, MO USA

**Keywords:** Ketamine, Depression, Functional connectivity, Limbic system, Subgenual anterior cingulate cortex, Hippocampus

## Abstract

Ketamine produces a rapid antidepressant response in over 50% of adults with treatment-resistant depression. A long infusion of ketamine may provide durable remission of depressive symptoms, but the safety, efficacy, and neurobiological correlates are unknown. In this open-label, proof-of-principle study, adults with treatment-resistant depression (*N* = 23) underwent a 96-h infusion of intravenous ketamine (0.15 mg/kg/h titrated toward 0.6 mg/kg/h). Clonidine was co-administered to reduce psychotomimetic effects. We measured clinical response for 8 weeks post-infusion. Resting-state functional magnetic resonance imaging was used to assess functional connectivity in patients pre- and 2 weeks post-infusion and in matched non-depressed controls (*N* = 27). We hypothesized that responders to therapy would demonstrate response-dependent connectivity changes while all subjects would show treatment-dependent connectivity changes. Most participants completed infusion (21/23; mean final dose 0.54 mg/kg/h, SD 0.13). The infusion was well tolerated with minimal cognitive and psychotomimetic side effects. Depressive symptoms were markedly reduced (MADRS 29 ± 4 at baseline to 9 ± 8 one day post-infusion), which was sustained at 2 weeks (13 ± 8) and 8 weeks (15 ± 8). Imaging demonstrated a response-dependent decrease in hyperconnectivity of the subgenual anterior cingulate cortex to the default mode network, and a treatment-dependent decrease in hyperconnectivity within the limbic system (hippocampus, amygdala, medial thalamus, nucleus accumbens). In exploratory analyses, connectivity was increased between the limbic system and frontal areas, and smaller right hippocampus volume at baseline predicted larger MADRS change. A single prolonged infusion of ketamine provides a tolerated, rapid, and sustained response in treatment-resistant depression and normalizes depression-related hyperconnectivity in the limbic system and frontal lobe.

ClinicalTrials.gov**:** Treatment Resistant Depression (Pilot), NCT01179009.

**Supplementary Information:**

The online version contains supplementary material available at 10.1007/s00213-021-05762-6.

## Introduction

Until recently, approved antidepressant medications have primarily targeted the brain monoamine systems (i.e., those involving serotonin, dopamine, norepinephrine). Monoaminergic antidepressant medications work slowly and require compliance, and, in many cases, are either not tolerated or not sufficient to produce remission of symptoms. The delayed therapeutic benefit of monoaminergic antidepressants has been linked to a slow increase in neurogenesis (particularly in the hippocampus) and transcriptional alterations that reconfigure neuronal circuits (Manji et al. 2001; Santarelli et al. 2003; Adachi et al. 2008). The limited targeting and slow rate of response of conventional antidepressants have motivated novel pharmacologic approaches.

Ketamine has generated substantial attention as a novel treatment for depression due to its rapid and robust antidepressant action. Ketamine is an antagonist of the NMDA subtype of the glutamate receptor, making it unique and novel among antidepressant medications. A brief 40-min infusion often provides relief to patients who have failed treatment with agents that target biogenic amines. While clinical response to ketamine can occur within hours, the antidepressant response often dissipates within 1 week in humans (Xu et al. 2016). Clinical trials of ketamine for chronic pain have reported that prolonged ketamine infusions over 4–14 days provide sustained relief of symptoms for as long as 8 months (Webster and Walker 2006; Sigtermans et al. 2009; Schwartzman et al. 2009; Niesters et al. 2014). This raises the question of whether a longer infusion paradigm might produce more durable antidepressant effects.

We recently demonstrated feasibility of a 96-h ketamine infusion for treatment-resistant depression (TRD) (Lenze et al. 2016). Rapid improvement was noted at 24 h for 7/10 subjects; moreover, the clinical response was sustained at 8 weeks in 4/10 subjects. Further investigation of treatment efficacy and underlying circuit mechanisms may aid in optimizing this novel intervention.

Given the neurochemical targets of ketamine and its rapid onset of action, there is broad interest in understanding its mechanism at molecular and neurocircuit levels (Krystal et al. 2019). Rodent models suggest that ketamine’s rapid antidepressant action arises through activation of the neurotrophic cascade; unopposed AMPA receptor signaling (Zanos 2016) activates a signaling pathway including brain-derived neurotrophic factor (BDNF), eukaryotic elongation factor 2 (eEF2), mechanistic target of rapamycin (mTOR), and glycogen synthase kinase-3 (GSK-3), which induces spine formation and synaptogenesis that is necessary and sufficient for antidepressant response (Li et al. 2010; Autry et al. 2011; Liu et al. 2013; Zhou et al. 2014; Moda-Sava et al. 2019). Interestingly, a similar phenomenon has been observed in treatment with serotonergic antidepressants (Adachi et al. 2008) and electroconvulsive therapy (Nibuya et al. 1995; Dukart et al. 2014), suggesting that the neurotrophic cascade may represent a common mechanism of antidepressant action (Duman and Monteggia 2006; Duman and Aghajanian 2012). Glutamatergic projections from limbic structures to the medial prefrontal cortex (Carreno et al. 2016; Farber 2019) also appear to be critical to antidepressant response to ketamine. However, the way in which these changes affect neural circuits involved in depression is not yet understood.

Functional connectivity (FC), measured using resting-state functional MRI (rsfMRI), is a tool for elucidating the changes in brain circuitry underlying antidepressant treatment. This technique has identified hyperconnectivity within the default mode network (DMN) as a cortical marker of depression (Kaiser et al. 2015). In particular, normalization of subgenual anterior cingulate cortex (sgACC) hyperactivity and hyperconnectivity has consistently been observed in antidepressant treatment (Siegle et al. 2012; Liston et al. 2014; Brown et al. 2020). The sgACC is a part of the DMN that receives direct inputs from the limbic system and plays a critical role in response to emotional stimuli (Freedman et al. 2000; Drevets et al. 1997). FC between the sgACC and other DMN regions correlates with depression severity (Greicius et al. 2007; Connolly et al. 2013), and normalization of FC is linked with antidepressant response across diverse treatment modalities (Dunlop et al. 2017), including ketamine (Gärtner et al. 2019). This suggests that sgACC FC may be an indicator of depression and recovery that is not treatment specific.

A small number of studies have specifically explored ketamine’s effects on human brain connectivity. Ketamine decreases FC between sgACC and DMN in non-depressed (Scheidegger et al. 2012) adults. In depressed individuals, ketamine reduced hyperactivity in the sgACC (Morris et al. 2020), and increased connectivity of some dorsolateral prefrontal regions (Abdallah et al. 2018; Gärtner et al. 2019). One recent study found significant changes in FC between the limbic and cortical executive networks in response to ketamine (Vasavada et al. 2020). Measuring blood oxygenation level-dependent signal in the hippocampus, amygdala, and thalamus is challenging due to methodological and logistical limitations of fMRI (Ojemann et al. 1997; Yan et al. 2013). Advances in image acquisition and processing have improved our ability to measure signal in these structures and enabled detailed assessment of limbic–cortico–striato–pallido–thalamic circuits (Greene et al. 2020). This is an important advance given that limbic structures are central to depression pathophysiology (Price and Drevets 2010) and to ketamine’s antidepressant effect.

Our goal in this open-label study was twofold. The first was to replicate and expand on our earlier clinical experience with 96-h ketamine infusions. The second was to use rsfMRI to assess potential neurobiological correlates of ketamine’s antidepressant effects. To accomplish this, we recruited 23 participants with treatment-resistant depression and administered a 96-h ketamine infusion. We used validated clinical assessments (MADRS, CGI-I) to assess clinical response, and we used rsfMRI at two timepoints before (to assess FC stability) and one timepoint after ketamine infusion to assess neurobiological mechanisms. Our hypothesis was that responders to ketamine would demonstrate response-dependent connectivity decreases in the sgACC and DMN, while all participants would show treatment-dependent connectivity changes in the limbic system.

## Methods and materials

### Enrollment

We enrolled adults ages 18–65 years with major depressive disorder. Confirmatory clinical evaluation was carried out by study psychiatrists, using a criterion of Montgomery-Asberg Depression Rating Scale (MADRS) (Montgomery and Åsberg 1979) score ≥ 22, to establish at least moderate symptom severity. We defined treatment resistance in the current episode as non-response to at least two trials of antidepressant medications of adequate dose and duration (Petersen et al. 2005). Continued use of selective serotonin reuptake inhibitors and serotonin-norepinephrine reuptake inhibitors was allowed if the dose was kept constant for at least 6 weeks leading up to the infusion.

Additionally, healthy subjects were enrolled and assessed at our center for the purposes of providing controls for imaging studies. We selected non-depressed individuals from that pool and then sub-selected to match them with the ketamine clinical trial participants on demographic (age, sex, race, and education) and imaging data quality (head motion, number of usable frames). Washington University’s institutional review board approved the study, and all participants gave written informed consent prior to any study procedures. See supplemental methods for exclusion criteria and matched control selection.

### Ketamine infusion and measurements

Participants were admitted for 5 days/4 nights at the Washington University Clinical Research Unit. Participants received a continuous 96-h infusion of intravenous ketamine, initiated at 0.15 mg/kg/h at 10 AM on day 1 and titrated as tolerated twice daily to a target rate of 0.6 mg/kg/h. The titration strategy and target infusion rate were based on anticipated plasma concentration of approximately 400 ng/ml (Newcomer et al. 1999; Goldberg et al. 2011) and an estimated 50% blockade of NMDA receptors (Hartvig et al. 1995; Emnett et al. 2013). All study participants were started on oral clonidine, 0.1 mg twice daily, approximately 7 days prior to the infusion, increased to 0.3 mg twice daily as tolerated, and stopped at completion of the infusion. See supplemental methods for details.

### Psychiatric and cognitive assessments

Study clinicians assessed psychotomimetic and other side effects of ketamine infusion using the Brief Psychiatric Rating Scale (BPRS)–positive symptom subscale (Flemenbaum and Zimmermann 1973), and an adverse events checklists and examination covering 20 ketamine side effects (Newcomer et al. 1999). Antidepressant response was assessed using the MADRS and the Clinical Global Impressions Improvement scale (CGI-I) (Guy 1976). All participants were assessed at 2, 4, 6, and 8 weeks post-infusion by phone with the MADRS and CGI-I (standard versions encompassing the previous 7 days). Psychometric research suggests that phone-based and in-person MADRS ratings are comparable (Kobak et al. 2008). See supplemental methods for details.

### Resting-state functional MRI acquisition and processing

Neuroimaging was performed on a Siemens Trio 3-T TIM scanner (Siemens, Erlangen, Germany). A gradient-recalled echo-planar sequence (EPI) was obtained while participants lay awake in the scanner performing no task. Two pre-infusion timepoints were acquired 1–14 days (median = 2 days) apart with the second scan occurring within 2 weeks of starting infusion. This was done to assess baseline FC variability and reliability. Two EPI runs, lasting 6 min each, were acquired at each of the three timepoints: the 2 pre-infusion and then at the 2-week post-infusion time point. At these three timepoints, 23, 18, and 20 subjects completed functional imaging. The control cohort was imaged on the Siemens Trio 3-T TIM scanner using an identical EPI protocol. After matching and exclusion, 27 control participants were included, 7 of which had two imaging timepoints.

Details on rsfMRI processing are provided in supplemental methods. Additional steps were taken to improve the measurement of functional connectivity in subcortical regions including multiple scans and methods to improve signal to noise ratio (see Additional fMRI Signal Cleanup and Subcortical Signal Analysis in supplement).

### Surface generation and brain areal parcellation

For cortical regions and resting state networks, we used a surface parcellation and community assignments generated by Gordon and Laumann and colleagues (Gordon et al. 2016). See supplemental methods for further details on surface generation and cortical parcellation.

For subcortical regions, we used a set of spherical regions of interest (Seitzman et al. 2020) generated to achieve full coverage and optimal region homogeneity. However, eight limbic regions (bilateral regions within the amygdala, anterior hippocampus proper, posterior hippocampus proper, and nucleus accumbens) were expanded to cover the entire anatomical structure (see Figs. 3d and S1). A subcortical limbic network was defined based on neuroanatomy: amygdala, antero-medial thalamus, nucleus accumbens, anterior hippocampus, posterior hippocampus (Price and Drevets 2010).

To generate region-wise connectivity matrices, time courses of all surface vertices or subcortical voxels within a region were averaged. Functional connectivity (FC) was then computed between each region timeseries using Fisher *z*-transformed Pearson correlation.

### Network FC analysis

Based on a review of the effects of both depression and ketamine on FC, we generated three a priori hypotheses: (1) FC within the DMN will decrease in patients whose depression improves after ketamine treatment (Scheidegger et al. 2012); (2) FC between the sgACC and DMN will decrease in patients whose depression improves after ketamine treatment (Greicius et al. 2007); and (3) FC within the limbic system will change after ketamine treatment (Price and Drevets 2010; McCabe and Mishor 2011). FC within the visual system was measured as a negative control in which no change was expected. Additionally, we divided participants based on treatment response at 2 weeks into responders (≥ 50% decrease in MADRS; *n* = 11) and non-responders (< 50% decrease in MADRS; *n* = 10).

We then conducted an exploratory FC analysis to visualize connectivity between the three a priori targets (DMN, sgACC, limbic system) and the rest of the brain. FC change was assessed by first measuring baseline FC by averaging the two timepoints acquired before ketamine (“Pre1” and “Pre2”) and then comparing baseline FC to the single timepoint acquired 2 weeks after ketamine infusion (“Post”).

### Volumetric analysis

We assessed the relationships between pre-ketamine volume of limbic structures (specifically, left and right hippocampus, amygdala, and nucleus accumbens) and response to ketamine. Specifically, pre-treatment volume, normalized by total intra-cranial volume, was correlated to change in MADRS score from baseline to 2 weeks post-infusion using a Pearson correlation; *p* values were not corrected for multiple comparisons in this exploratory analysis.

### Statistical approach

Primary outcomes for this study were improvements in MADRS relative to baseline, and CGI-I—specifically, number of patients scoring “much” or “very much improved.” An ANOVA with time as a repeated measure was used to assess significance. Post hoc comparisons with a paired *t* test were used to assess the effect at different time points when the overall ANOVA was significant. Rate of response (defined as MADRS reduction of at least 50% from baseline) and rate of remission (defined as MADRS < 10) were chosen as secondary outcomes. Comparisons were made to the “baseline” timepoint. Outcomes were interpreted using a per-protocol analysis.

MADRS change was also compared to other variables (e.g., serum concentrations of total ketamine, s- and r-ketamine, s- and r-norketamine, total norketamine, BPRS positive scale averaged across infusion days) using Pearson correlations. A logistic regression was used to assess relationship between ketamine metabolite blood concentrations and side effects.

For functional connectivity analyses, FC data from depression subjects were entered into a linear mixed effects model to assess the relationships between time (before versus after treatment; independent variable), treatment response (responder versus non-responder; independent variable), and functional connectivity (dependent variable). Time and an interaction of time by response were treated as fixed effects. Participant was treated as a random effect to account for individual differences. In total, 21 subjects with 40 observations (two were missing data after treatment) were included in the model. Seed maps demonstrating brain-wide connectivity of the sgACC and limbic regions were for visualization only and no statistical tests were done.

In total, eight primary outcomes (two clinical + six neuroimaging) were chosen. Thus, strict (Bonferroni) correction for multiple comparisons would set the threshold of *p* < 0.00625. Statistics are reported as raw (uncorrected) *p* values generated by their respective model, but we also report which primary outcome measures do not pass significance after Bonferroni correction.

To better interpret effects of intervention on brain network connectivity, we conducted a post hoc comparison of FC between matched controls and depression subjects (at baseline and after ketamine) for the three hypothesized systems above plus the visual system (negative control). This was done by averaging FC data across timepoints when a participant had two timepoints in the same condition (i.e., for patients’ two baseline imaging visits) and then conducting unpaired two-tailed *t* tests (eight in total) between controls and patients at baseline and again between controls and patients after ketamine. Statistics for this post hoc analysis are reported as raw (uncorrected) *p* values.

## Results

### Prolonged ketamine infusion is tolerated and produced target drug concentrations

From January 2014 to August 2016, we consented 27 participants via referrals and clinicaltrials.gov: 1 failed to meet inclusion criteria and 3 withdrew consent prior to the infusion, leaving 23 eligible participants. Table 1 shows the baseline characteristics of the 23 participants who initiated infusion. The infusion procedure was well-tolerated, with all but one of the individuals completing the infusion. Another patient was lost to follow-up after completing the “day 6” follow-up. Of the 22 participants who completed the 96-h infusion, 5 participants did not tolerate the maximum target infusion rate, primarily due to nausea and anxiety. The mean final dose was 0.54 mg/kg/h (SD = 0.13). Concentrations of ketamine and its major metabolites obtained over the course of treatment are listed in Table S1.

**Table 1:** Patient characteristics

Patient Characteristics	mean (SD)
Age	40.0 (14.05)
Sex, n (%)	
Male	13 (56.52)
Female	10 (43.48)
Race, n (%)	
White	23 (100%)
Non-White	0 (0%)
Ethnicity, n (%)	
Hispanic/Latino	0 (0%)
Not Hispanic/Latino	22 (96%)
Unknown/Not Reported	1 (4%)
Severity of Illness, n (%)	
Moderately ill	7 (30%)
Markedly ill	15 (65%)
Severely ill	1 (4%)
Previous Antidepressant Trials	8.2 (5)
Total MADRS Score (baseline)	28.83 (3.94)
Max Tolerated Dose (mg/kg-hr)	0.54 (0.13)

Side effects were generally mild in intensity, improved over the course of the infusion, and subsided quickly after stopping infusion (Table S3). At time of discharge, some participants still reported fatigue (14%, 3/21), inattention (14%, 3/21), sedation (14%, 3/21), lightheadedness (5%, 1/21), restlessness (5%, 1/21), and palpitations (5%, 1/21). These are comparable to symptoms reported prior to starting the infusion. On average, there was minimal increase in systolic (8 mm Hg) and diastolic blood pressure (5 mm Hg) at 8 AM on day 5 compared to pre-infusion (Fig. S2). Psychotomimetic effects (BPRS positive scale) were mild (Fig. S3), peaked on infusion day 3 (mean 6.3 ± 1.58), and tended to decline during infusion days 4–5. Side effects were not correlated with serum concentrations of ketamine or any of its metabolites (data not shown).

### Prolonged ketamine infusion induced persistent improvements in depression severity

**Fig. 1:**
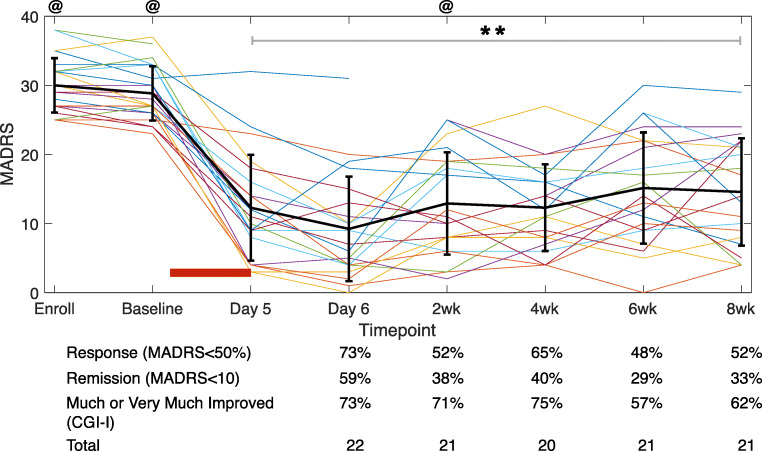
Clinical response to 96 h ketamine infusion. MADRS scores were significantly improved by ketamine (repeated measures ANOVA *p* < 0.001). Mean MADRS was 29 pre-infusion, 9 one day post-infusion, 13 and 15 at 2 and 8 weeks post-infusion, respectively. Black line shows mean and SD. Double asterisk indicates *p* < 0.001 for all post-infusion timepoints versus baseline. Red bar indicates infusion period; @ indicates neuroimaging timepoints. Below, percent of patients meeting criteria for response, remission, and CGI-I of 1-2 (much or very much improved), as well as total number of subjects at each timepoint

MADRS scores fell significantly after the ketamine infusion ended and then rose slowly over the next 8 weeks (Fig. 1). Mean MADRS was 29 (SD = 4) pre-infusion. Post-infusion, mean MADRS was 9 at one day (SD = 8, *p* < 0.001; Cohen’s d = 1.7), 13 at 2 weeks (SD = 8, *p* < 0.001; d = 1.6), and 15 at 8 weeks (SD = 8, *p* < 0.001; d = 1.5). One day after infusion ended, 73% (16/22) of subjects showed response (at least a 50% reduction in MADRS) (Fig. 1) with 59% (13/22) in remission (MADRS < 10). At 2 weeks, 52% (11/21) of subjects still showed response, with 38% (8/21) in remission. At 8 weeks, results were similar to the 2-week timepoint: 52% (11/21) of subjects still showed response, with 33% in remission. Of those who responded at 2 weeks, 73% (8/11) remained responders at 8 weeks. The only patient who continued to have MADRS > 30 on post-infusion day 1 missed subsequent timepoints due to psychiatric hospitalization. If we assumed that this patient remained severely depressed, then response rates at 2 and 8 weeks would be 50% (11/22). Consistent with prior reports (Luckenbaugh et al. 2014; Pennybaker et al. 2017), participants’ BPRS positive scale scores (averaged across infusion days) showed an inverse relationship to MADRS change at 1 day post-infusion (r = − 0.54, *p* = 0.009), but the relationship was no longer significant by 2 and 8 weeks (both *p* > 0.1; Fig. S3). CGI-I scores paralleled MADRS changes: 73% (16/22) were much improved or very much improved 1 day post-infusion, while 71% (15/21) and 62% (13/21) were much improved or very much improved at 2 and 8 weeks, respectively (Fig. 1).

Response to treatment at 2 weeks was not significantly correlated with mean serum concentration of ketamine (Fig. S3) or its major metabolites s- and r-norketamine (*p* > 0.05).

### Effects of ketamine on DMN FC

We used rsfMRI to measure the sustained effects of ketamine on brain connectivity, based on FC measures taken before and 2 weeks after ketamine infusion. After strict imaging exclusion criteria, 19 subjects had at least one pre-scan and post-scan timepoint.

We first assessed change in DMN FC (averaged across all DMN node pairs) in response to ketamine, using a linear mixed-effects model. Connectivity within the DMN decreased 2 weeks after ketamine infusion (*p* = 0.003, T = − 3.2, DF = 37; Fig. S4A–C). The interaction of time and response yielded a *p* value of 0.051 (T = 2.0, DF = 37). In other words, responders (≥ 50% decrease in MADRS at 2 weeks; *n* = 11) showed a trend toward larger decreases in DMN connectivity than non-responders (*n* = 10; Fig. S4E).

### Ketamine reduces subgenual anterior cingulate cortex to DMN FC

**Fig. 2:**
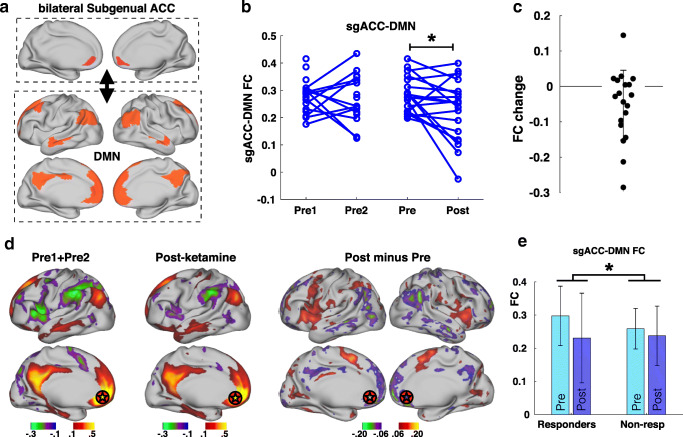
Connectivity between subgenual anterior cingulate (sgACC) and Default Mode Network (DMN) decreases with ketamine infusion. **a** Regions included in bilateral sgACC and DMN are shown. **b** Blue lines on the left represent normal variability of FC between the sgACC and DMN in depressed patients (open circles are individuals with a single pre timepoint). Blue lines on the right represent change in FC resulting from ketamine infusion. Asterisk indicates that main effect of time (pre-infusion vs post-infusion) was significant (*p* = 0.001). **c** FC change in TRD patients (post-infusion minus pre-infusion). Error bars = SD of FC change. **d** Maps of cortical FC of bilateral sgACC (target symbol) before and after ketamine. **e** Comparison of FC change between responders (greater than 50% reduction in MADRS) and non-responders (less than 50% reduction in MADRS). Asterisk indicates the interaction of time-by-response was significant (*p* = 0.028). Error bars = SD

We next tested the narrower hypothesis that FC between the bilateral sgACC and the rest of the DMN would decrease in response to ketamine. Here, we found an effect of time and an interaction of time-by-response. Connectivity between sgACC and the remainder of the DMN decreased 2 weeks after ketamine infusion (*p* = 0.001, T = − 3.5, DF = 37; Fig. 2a–c). In response to ketamine, sgACC connectivity decreased to major components of the DMN across cortical hemispheres (Fig. 2d). There was a concurrent increase in FC between sgACC and other frontal areas, particularly the bilateral caudal anterior cingulate and bilateral anterior insula. In addition, responders showed a larger decrease in sgACC-DMN connectivity than non-responders (Fig. 2e; *p* = 0.028, T = 2.3, DF = 37). However, this does not pass Bonferroni-corrected threshold of *p* < 0.00625.

### Ketamine reduces connectivity within the limbic system

Limbic regions, nucleus accumbens and amygdala in particular, have lower raw rsfMRI signal and higher noise than cortex. In these data, signal in limbic regions was substantially better than cortical areas known to have signal dropout (Fig. S7). Following our image processing approach (see Additional fMRI Signal Cleanup), SD in limbic structures was no different from cortical regions, suggesting that additional sources of noise affecting limbic signal had been removed (Fig. S7B). Baseline FC values within the limbic network were around *r* = 0.2 on average (Fig. 2b; roughly the same as FC values in the cortical visual and ventral attention networks). Limbic regions showed FC with sgACC and other parts of the DMN (Fig. S6A).

**Fig. 3:**
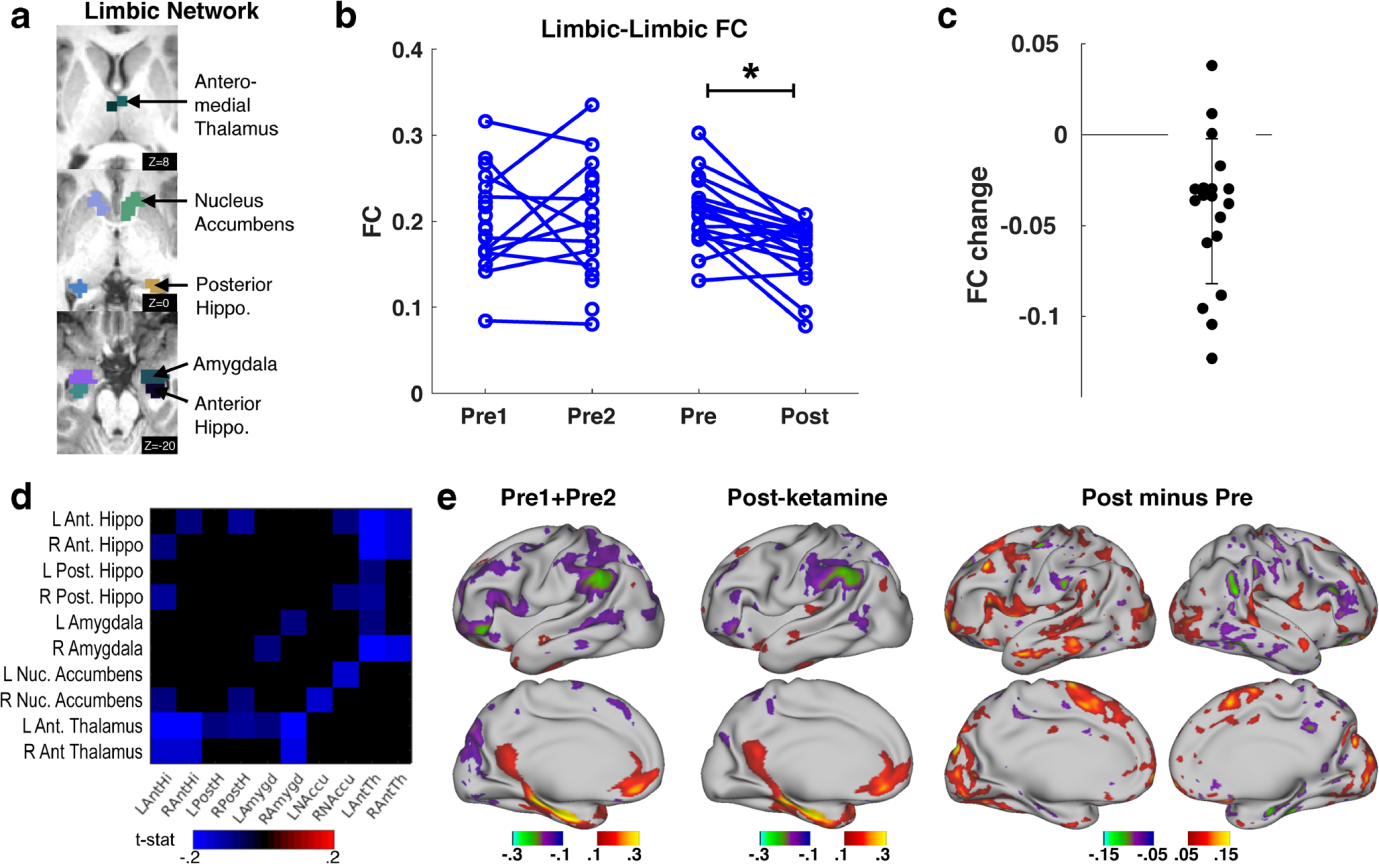
Connectivity within the limbic system decreases after ketamine infusion. **a** Regions of interest included in the limbic network. **b** Blue lines on the left represent normal variability of limbic FC (open circles are individuals with a single pre timepoint, black lines are mean of patients with both timepoints). Blue lines on the right represent change in FC resulting from ketamine infusion. Asterisk indicates that main effect of time (pre-infusion vs post-infusion) was significant (*p* = 0.003). **c** FC change in TRD patients (post-infusion minus pre-infusion). Error bars = SD of FC change. **d** Matrix of FC change between limbic regions. Colored squares in the matrix indicate pairs of limbic regions whose connectivity changes as a result of ketamine infusion (red indicates increased FC, blue indicates decreased FC). The matrix shows t-statistics thresholded by *p* < 0.1. **e** Group-average seed FC between limbic network and cortical regions before and after ketamine are projected on the right hemisphere. The difference map is also shown for both hemispheres

Ketamine infusion caused a decrease in FC within the limbic system of depressed individuals (*p* = 0.003, T = − 3.2, DF = 37; Fig. 3a–c). By comparison, signal and noise properties of limbic regions did not change (Fig S7). Ketamine-induced change in limbic FC did not differ between responders (≥ 50% decrease in MADRS) and non-responders (*p* = 0.54, T = 0.61, DF = 37; Fig. S5). While connections between nearly all parts of the limbic system decreased after ketamine, FC between the anterior thalamus and other limbic structures showed particularly a large decrease (Fig. 3d). When FC between limbic regions and the cortex was visualized, we observed increased FC to many cortical areas after treatment, particularly frontal components of the cingulo-opercular network (Fig. 3e; Fig. S6B).

### Depression-related hyperconnectivity in the default network and limbic system

**Fig. 4:**
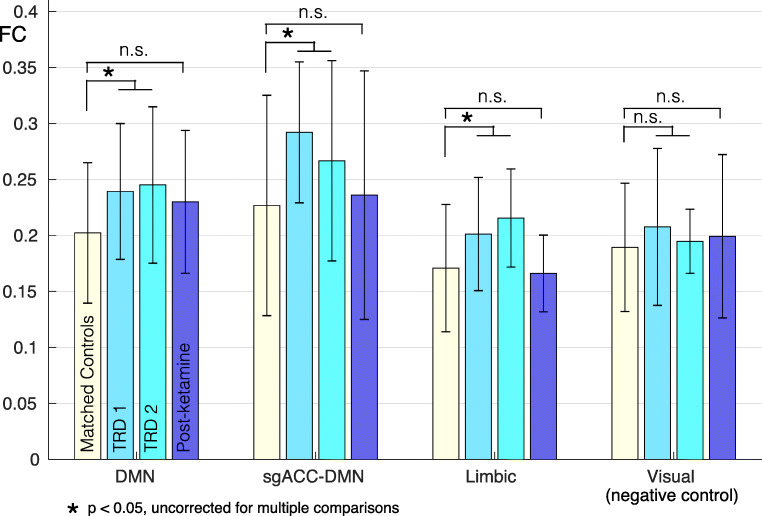
Depression-related hyperconnectivity is seen in the three hypothesized systems, but not the visual system. The four clusters of bars indicate group mean and SD of functional connectivity within (1) the entire DMN, (2) subgenual anterior cingulate to DMN, (3) the limbic system, and (4) the visual system for matched controls (cream) and for patients before (cyan, two timepoints), and after ketamine treatment (blue). Black asterisks indicate a difference (2-tailed *t* test; *p* < 0.05 uncorrected) between groups. Error bars = SD. sgACC, subgenual anterior cingulate cortex; DMN, default mode network

To better interpret the changes in functional connectivity observed after ketamine, we compared the TRD subjects to healthy matched controls. We found depression-related hyperconnectivity in the hypothesized systems (limbic system, default mode network, and a subset of default connections with the subgenual anterior cingulate) in pre-treatment TRD subjects relative to controls (Fig. 4; 2-tailed *t* test; *p* < 0.05 uncorrected). No difference between patients and controls was seen in the visual system. No difference was seen between healthy controls and post-ketamine TRD patients in any of the four systems tested.

### Right hippocampus volume predicts ketamine response

**Fig. 5:**
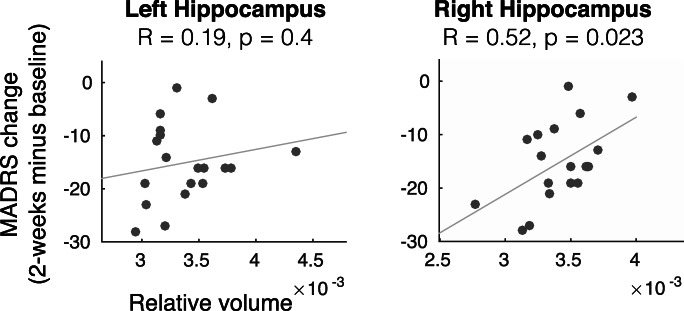
Pre-treatment hippocampal volume predicts treatment response. The x-axis depicts volume of the hippocampus as a proportion of intracranial volume; the y-axis reflects change in MADRS score from baseline to two weeks post-infusion. *p* values are not corrected for multiple comparisons (6 comparisons done)

In addition, we explored the association between limbic structural volumes and clinical response to ketamine (6 structures—left and right hippocampus, amygdala, and NAcc). We observed a correlation between right hippocampal volume and MADRS score reduction (i.e., smaller pre-treatment right hippocampal volume predicted greater improvement in response to ketamine) (Pearson’s *r* = 0.52, *p* = 0.023 uncorrected) (Fig. 5). None of the five other limbic structures tested showed a significant relationship.

## Discussion

### Long infusion paradigm

Our examination of clinical and neuroimaging outcomes of a 96-h, high-dose ketamine infusion for adults with treatment-resistant depression provided two key observations. The first is that a single, prolonged, high-dose infusion may offer more persistent antidepressant effect than brief ketamine bolus. The response rate at 2 weeks post-infusion (52%, 11/21) is similar to the response rate that has been reported at 4 weeks with recurrent twice-weekly intranasal esketamine of 50–55% in ketamine-naive treatment-resistant depression patients (Fedgchin et al. 2019). However, in the present study, treatment response remained unchanged at 8 weeks without any further treatment (52%, 11/21). This is consistent with our previous 96-h ketamine infusion feasibility study (Lenze et al. 2016) and with trials of long ketamine infusions for chronic pain that have reported responses lasting many months (Webster and Walker 2006; Sigtermans et al. 2009; Schwartzman et al. 2009; Niesters et al. 2014). By contrast, antidepressant effects of a single 40-min ketamine bolus tend to dissipate after around 1 week (Xu et al. 2016). Taken together, this evidence indicates that a longer infusion may extend the antidepressant effects of ketamine. A placebo-controlled study will be needed to validate our assertion of a prolonged response.

It may be that a higher total ketamine dose and a longer period of NMDA receptor blockade reduce the relapse rate following cessation of treatment. Animal studies have shown that ketamine bolus increases BDNF RNA translation but not transcription (Autry et al. 2011). Thus, it is possible that the longer infusion is needed to induce transcriptional change.

The second key finding from this study is that subjects demonstrated normalization of depression-related hyperconnectivity following high-dose ketamine infusion. Specifically, participants showed a decrease in FC between the subgenual anterior cingulate and DMN (that was larger in responders), and within the limbic system (that was independent of clinical response). There were also FC changes between limbic and other brain areas. This study is the first to directly investigate the effects of ketamine on the limbic system in humans suffering from treatment-resistant depression, and it points to potential mechanisms underlying ketamine’s antidepressant effects.

### Subgenual cingulate and DMN

The observed decrease in DMN connectivity after ketamine may reflect normalization of hyperconnectivity that has been linked to pathological introspection and rumination in depression (Hamilton et al. 2015). We focused on sgACC connectivity in particular because it is perhaps the most robust marker of both depression severity and treatment and response. Depression has consistently been associated with sgACC volume (Drevets et al. 1997; Bora et al. 2012), hyperactivity (Drevets et al. 2008; Siegle et al. 2012), and hyperconnectivity with the DMN (Greicius et al. 2007) of the sgACC. We observed that ketamine infusion produced a decrease in FC between bilateral sgACC and the DMN. This parallels previous studies measuring the effects of SSRI (Dunlop et al. 2017) and TMS (Liston et al. 2014). We, therefore, interpret the observed decrease in FC to reflect a general effect of recovery from depression. This is supported by the observation that responders showed a larger decrease in sgACC-DMN FC than non-responders. By observing this treatment non-specific phenomenon, we view this as a positive control for our subsequent FC analysis.

### Limbic system

We found effects of ketamine on FC within the limbic system (Figs. 4, S6), particularly the anterior thalamus and anterior hippocampus. This finding is consistent with preclinical studies suggesting works by activating the neurotrophic cascade in the limbic system (Autry et al. 2011; Zanos et al. 2016; Krystal et al. 2019) but it has not been reported in prior human ketamine FC studies. This may be because of methodological advances taken here to improve signal-to-noise in these regions. In contrast to within-DMN and sgACC-DMN FC, we argue that limbic FC change may be a ketamine-specific effect. Vasavada and colleagues recently reported FC changes between hippocampus, amygdala, and executive control networks 24 h after a short ketamine infusion (Vasavada et al. 2020). Our results partly complement (Fig. S6) and extend those by showing large FC decreases within the limbic system and in particular between the anterior thalamus and other limbic structures.

Past neuroimaging studies have found limbic FC to correspond to stress-related measures. For example, amygdala-hippocampus FC positively correlates with psychosocial stress and history of abuse (Fan et al. 2015), and increases transiently (for hours) after an acute social stressor (Vaisvaser et al. 2013). These effects are mediated by the glucocorticoid system (Hall et al. 2015). Relatedly, prior work using magnetoencephalography found that response to fearful faces predicted antidepressant response to ketamine (Salvadore et al. 2009). Thus, it is possible that the observed reduction in limbic FC reflects a modulation in limbic stress response after ketamine.

Animal studies have also suggested that ketamine’s antidepressant effects depend on activation of hippocampal-prefrontal projections (Carreno et al. 2016). We observed increased FC between limbic regions and the anterior cingulate cortex (Fig. S6), suggesting that this mechanism may be conserved in humans. However, it should be noted that the observed hippocampal-frontal FC increase was actually a loss of pre-treatment anticorrelation, making interpretation difficult.

Finally, the observed relationship between right hippocampal volume and ketamine response in our exploratory analysis is of potential clinical importance. Similar to previous results (Abdallah et al. 2015, 2017), we observed that smaller hippocampal volume predicted better response to ketamine. Reduced hippocampal volume has been associated with a number of psychiatric disorders, most notably major depressive disorder (Videbech and Ravnkilde 2004; Schmaal et al. 2016). Conversely, a number of studies have reported that smaller pre-treatment hippocampal volume predicts worse response to serotonergic antidepressants (Colle et al. 2018). Thus, hippocampal volume may be of clinical value in choosing between treatment strategies (serotonergic antidepressant versus ketamine).

## Limitations and conclusions

The greatest limitation of this study is that no placebo condition was used. Without an active placebo, such as short infusion of ketamine or other CNS active agent, it is not possible to determine how other aspects of the clinical trial (such as intensive monitoring) affected clinical response. This limits our ability to make strong conclusions about the clinical efficacy of 96-h ketamine infusions. However, response data here are similar to that seen in our prior 96-h ketamine trial that utilized a 40-min ketamine infusion control group (Lenze et al., 2016) and to that seen in other ketamine studies, suggesting that the observed response is not an artifact. Similarly, it cannot be said with certainty if observed connectivity changes are attributable to ketamine or placebo effect. Now that potential clinical benefit and neuroimaging correlates have been found with the 96-h ketamine infusion paradigm, a randomized controlled clinical trial to confirm these results is needed.

Future studies are also needed to determine optimal parameters of treatment and to understand clinical outcomes. This paradigm was prompted by studies in chronic pain showing that 96-h infusion produces a clinical response sustained for as long as 8 months (Niesters et al. 2014). But it is possible that a much shorter infusion might have the same effect. Thus, it will be important to vary infusion duration and follow patients for a longer period of time to better understand duration of clinical benefit. Moreover, while it has been demonstrated that clonidine reduces psychotomimetic effects of ketamine (Farber et al. 1995; Sollazzi et al. 2009), further work is needed to determine what effect clonidine has on the antidepressant properties of ketamine.

Another limitation is the barrier to clinical adoption of a 96-h infusion. Specifically, cost and logistics may make such a long infusion unrealistic for an outpatient population, who might prefer serial twice-weekly ketamine boluses. Even so, it may be practical to provide a long duration infusion to hospitalized patients, as has been reported in previous studies of long ketamine infusion for patients with chronic pain (Niesters et al. 2014) and more recently with brexanolone 60-h infusions in post-partum depression. A goal of further clinical development that may mitigate those barriers would be to optimize patient selection via an enrichment strategy. For example, patients could first receive a brief ketamine dose, and then those who respond clinically would be considered for a 96-h infusion (Lenze et al. 2020). Similarly, future work could identify and validate neurobiological predictors of therapeutic response (such as hippocampal volume). Given the novel mechanism of ketamine, these neurobiological questions may help not only to optimize ketamine therapy but also to guide the search for novel interventions for depression (Heifets and Malenka 2019).

## Supplementary Information

ESM 1 (DOCX 4589 kb)
