# Supplementary material for: Prolonged ketamine infusion modulates limbic connectivity and induces sustained remission of treatment-resistant depression
Source: Psychopharmacology (Berl). 2021 Jan 22;238(4):1157–69. doi: 10.1007/s00213-021-05762-6 (PMC7969576; doi:10.1007/s00213-021-05762-6)
Supplement: Supplementary file 1 — (DOCX 4589 kb) [file 213_2021_5762_MOESM1_ESM.docx]

**Prolonged ketamine infusion modulates limbic connectivity and induces sustained remission of treatment-resistant depression**

***Supplemental Information***

## General inclusion/exclusion criteria (for both depression and control groups)

General inclusion criteria: education level >8 years, ability to read and write in English, able to provide informed written consent. General exclusion criteria: neurological disorders that may confound results (stroke, epilepsy, etc.), bipolar disorder, lifetime psychotic disorder, substance abuse/dependence, substance use (confirmed by urine drug screen, if tested positive for marijuana and did not appear impaired, we usually let them continue with the study that day) and medical instability.

## Depression inclusion/exclusion criteria and Enrollment

Participants were recruited via referrals and clinicaltrials.gov. Major depressive disorder was diagnosed using the Diagnostic Interview for Genetic Studies (DIGS) [1]. Confirmatory clinical evaluation was carried out by study psychiatrists, using a criterion of Montgomery-Asberg Depression Rating Scale (MADRS) [2] score ≥22, to establish at least moderate symptom severity. We defined treatment resistance in the current episode as non-response to at least two trials of antidepressant medications of adequate dose and duration [3].

Medication-related exclusions included dopamine agonists, antagonists or reuptake inhibitors (other than aripiprazole at doses ≤5mg daily); centrally acting pro- or anti-cholinergics; or benzodiazepines or other GABA-acting agents. Patients taking these medications were excluded based on the knowledge that NMDA antagonists abolish GABAergic inhibition, resulting in the excessive release of acetylcholine and glutamate [4]. This process is linked to the psychotomimetic effects of ketamine, though it is unclear how it relates to the antidepressant effects.

Enrollment of depression participants was stopped after N=20 with pre- and post-ketamine neuroimaging data, which was predetermined based on feasibility goals.

## Matched control inclusion/exclusion criteria

Non-depressed control participants were recruited from Washington University (ages 18-25) and from flyers and ads distributed in the St. Louis community and the Research Participant Registry (all ages) between the years of 2009-2016. Control participants were excluded for history of traumatic brain injury with loss of consciousness >30 minutes. Because these participants were also recruited as controls for HIV studies, HIV-negative status was confirmed at the time of study using a buccal swab. Typically, the consent, questionnaires, cognitive testing, and MRI scan all occurred on the same day. Some participants were brought in for a second session to enable assessment of test-retest variability.

## Matched control selection

A subset of controls was selected from a pool of 150 unique individuals to match for demographics and data quality using the following steps. First, any controls with at least mild depression symptoms (Beck Depression Inventory > 9) were excluded, leaving 106 unique control subjects. Of those, a subset of controls was then chosen to match to the ketamine TRD cohort based on 1) age, 2) gender, 3) race, and 4) education, leaving 43 unique control subjects. This cohort were average age 34.2 ±16.7, 23/43 male, and 100% non-hispanic.

Controls were then excluded for head motion and amount of usable resting fMRI data, leaving a final matched cohort of 27 control subjects, 7 of which had two usable imaging timepoints. Controls were well matched to patients on head motion parameters (Table S2).

## Ketamine blood level monitoring

Venous blood was obtained daily, centrifuged, plasma stored at -20°C, and later assayed for enantiomeric (i.e., R- and S-) ketamine and active metabolite (norketamine) concentrations at 10am on days 2-5. R- and S-ketamine and R- and S-norketamine were determined by HPLC-tandem mass spectrometry using a previously published method ([Moaddel et al., 2010](#_ENREF_7)).

## Clonidine

All study participants were started on oral clonidine 0.1mg twice daily, approximately seven days prior to the infusion. Oral clonidine was increased as tolerated to 0.2 mg twice daily starting on the evening of day 1 of the infusion, and then 0.3mg twice daily starting on the evening of day 2. The decision to co-administer clonidine, an alpha-2 agonist, was based on research on ketamine and other NMDA antagonists (Farber et al. 1995) showing that cholinergic inputs from the basal forebrain onto corticolimbic neurons become hyperactive with systemic NMDA blockade and this phenomenon may contribute to dissociative effects of ketamine [5,6]. Thus, clonidine is theorized to block psychotomimetic (dissociative) and sympathomimetic (blood pressure) effects of ketamine but not antidepressant effects [7]. Ketamine and clonidine were stopped on the morning of day 5. Participants were discharged approximately four hours post-infusion and stable vitals.

|  | s-ketamine Mean (ng/ml) | r-ketamine Mean | total ketamine Mean | s-nor-ketamine Mean | r-nor-ketamine Mean | total nor-ketamine Mean |
| --- | --- | --- | --- | --- | --- | --- |
|  |  |  |  |  |  |  |
| Infusion Day 1 | 54.8 | 65.1 | 119.9 | 38.9 | 44.7 | 83.6 |
| Infusion Day 2 | 174.2 | 192.8 | 367.0 | 151.0 | 171.0 | 322.0 |
| Infusion Day 3 | 214.1 | 237.9 | 452.0 | 162.1 | 202.5 | 364.6 |
| Infusion Day 4 | 217.7 | 242.5 | 460.2 | 144.7 | 177.3 | 322.0 |
| Infusion Day 5 | 242.6 | 272.5 | 515.0 | 145.6 | 185.8 | 331.4 |

**Table S1. Ketamine concentrations.** Average serum concentrations based on venous blood samples collected at 10AM on each day.

## Psychiatric and Cognitive Assessments

We assessed cognitive and behavioral effects of the ketamine infusion in two ways. First, participants were assessed daily with the Brief Psychiatric Rating Scale ([Flemenbaum and Zimmermann, 1973](#_ENREF_3)) four-item positive symptom subscale (BPRS+; scale range 4-28, higher scores indicate greater psychotic symptoms; items are Conceptual Disorganization, Suspiciousness, Hallucinatory Behavior, and Unusual Thought Content), as a scale of psychotomimetic symptoms. Second, participants were assessed with the clinician-administered Clinical and Adverse Events Checklist of 20 ketamine side effects using a 5-point Likert scale (0 = symptom absent, 1= minimal, 2= mild, 3 = moderate, 4= severe) ([Newcomer et al., 1999](#_ENREF_9)), up to four times daily, to measure psychotomimetic, cognitive and other behavioral or physical symptoms. Vital signs were measured via continuous electrocardiographic monitoring as well as blood pressure monitoring hourly for 4 hours after ketamine initiation and dose augmentation; blood pressure was also measured every 8 hours.

During ketamine infusion, depressive symptoms were assessed daily with a 24-hour version of the MADRS, with modifications based on a published structured interview ([Williams and Kobak, 2008](#_ENREF_15)). One day post-infusion, the 24-hour MADRS and the Clinical Global Impressions Improvement scale (CGI-I) [8] were conducted to examine rapid antidepressant effects. All participants were assessed at 2, 4, 6, and 8-weeks post-infusion by phone with the MADRS and CGI-I (standard versions encompassing the previous 7 days).

## Resting-State Functional MRI Processing and Surface Projection

Neuroimaging was performed on a Siemens Trio 3T TIM scanner (Siemens, Erlangen, Germany) in the MIR Center for Clinical Imaging Research located at the Washington University Medical Center. A gradient recalled echo-planar sequence (EPI) [Repetition time [TR]=2200ms, echo time [TE]=27ms, flip angle=90°, 4x4x4 mm voxels] was used to capture images of blood oxygenation level-dependent (BOLD) contrast responses. Participants were instructed to remain awake in the scanner performing no task (eyes open, no music, headphones in place). EPI images of the whole brain were volume acquisitions across 36 axial slices. For depression participants, two EPI runs, lasting 6 minutes each, were acquired at each of three timepoints: twice pre-infusion and at 2 weeks after infusion. At these three timepoints, 23, 18, and 20 subjects completed functional imaging. Control participants were imaging on the same scanner using the same protocol. A subset was brought back for a second timepoint to assess test-retest reliability. All steps described below apply to both cohorts.

Preprocessing of fMRI data included: 1) compensation for asynchronous slice acquisition using sinc interpolation; 2) elimination of odd/even slice intensity differences resulting from interleaved acquisition; 3) whole brain intensity normalization to achieve a mode value of 1000; 4) removal of distortion using field map and spatial realignment within and across fMRI runs; 5) resampling to 3mm cubic voxels in atlas space including nonlinear realignment and atlas transformation in one resampling step. Cross-modal (e.g., T2-weighted to T1-weighted) image registration was accomplished by aligning image gradients [9].

Following cross-modal registration, data were passed through several additional preprocessing steps: (i) tissue-based regressors were computed based on FreeSurfer segmentation [10]; (ii) removal by regression of the following signals that contain spurious variance: (a) six parameters obtained by rigid body correction of head motion, (b) the signal averaged over the whole brain, (c) signal from white matter, ventricles and extra-axial sources of noise (see **Additional fMRI Signal Cleanup**); (ii) temporal filtering to retain frequencies in the 0.009–0.08-Hz band; and (iii) frame censoring. The first four frames of each BOLD run were excluded. As has been reported previously, several subjects exhibited high-frequency peaks in the power spectrum of head motion time courses, primarily in the phase-encoding (y) dimension. Thus, we low-pass filtered the motion time courses at 0.1Hz in all subjects prior to computing FD to prevent superfluous data loss [11,12]. Frame censoring was implemented using framewise displacement [13] with a threshold of 0.13 mm. This frame-censoring criterion was uniformly applied to all rsfMRI data before functional connectivity computations. FC quality-based exclusion criteria included 1) less than 164 (50%) usable frames after motion scrubbing, and 2) severe aberrancy in BOLD signal SD (greater than 3 SD outside of group median), and 3) study completion.

|  | Controls Mean(SD) | TRD Baseline1 Mean(SD) | TRD Baseline 2 Mean(SD) | TRD Post-Ket Mean(SD) |
| --- | --- | --- | --- | --- |
| Framewise Displacement (unscrubbed) | 0.177(.086) | 0.164(.052) | 0.198(0.089) | 0.195(0.073) |
| Frames Acq. | 328(0) | 328(0) | 328(0) | 328(0) |
| % Frames Retained after scrubbing | 87(13) | 92(4) | 88(12) | 87(9) |

**Table S2.**

Individualized cortical surfaces and subcortical volumes were generated for each subject’s T1 MRI using FreeSurfer automated segmentation [10]. Segmentation errors were manually corrected. Following preprocessing, BOLD data were sampled to each subject’s individual cortical surface and subcortical volume using Connectome Workbench [14].

Brain surface visualizations were generated using Connectome Workbench [14].

## Additional fMRI Signal Cleanup

Advances in image acquisition and processing have improved our ability to measure signal, particularly in subcortical structures [15–18]. A number of steps were taken to improve signal to noise ratio in subcortical regions. First, nuisance regressors were extracted from individualized white matter, and ventricles (defined via FreeSurfer segmentation). Next, because voxels on the edge of the brain are particularly susceptible to artifacts from motion, vasculature, and CSF [19,20], a set of extra-axial regressors was generated by thresholding the temporal standard deviation image (temporal SD > 2.5%) and excluding a dilated whole brain mask [16,17]. Voxel-wise nuisance time series were dimensionality reduced as in CompCor [16], except that the number of retained regressors, rather than being a fixed quantity, was determined, for each noise compartment, by orthogonalization of the covariance matrix and retaining components based on eigenvalues with condition number > 30 (${\lambda_{max}}/{\lambda_{min}}>30$). This was done because number of noise-related components seems to differ across subjects.

During surface projection, an additional step was taken to remove sources of noise potentially related to the presence of nearby blood vessels [18]. Voxels with a high coefficient of variation (0.5 standard deviations above the mean coefficient of variation of all voxels in a 5mm sigma Gaussian neighborhood) were excluded (this is referred to as a ‘goodvoxels’ mask) and missing timeseries were interpolated based on neighbors.

## Surface Generation and Brain Areal Parcellation

Surface generation and processing of functional data followed procedures similar to [18]. For cortical regions and resting state networks, we used a surface parcellation and community assignments generated by Gordon & Laumann and colleagues [21]. Based on that parcellation (Figure S1), the following cortical resting-state FC networks were defined using infomap: Salience (black); Visual network (blue); Default Mode Network (red); Dorsal Attention (green); Ventral Attention/Language (teal); Congulo-opercular (purple); dorsal somato-motor (sky blue); ventral somato-motor (orange); auditory (pink); frontoparietal network (yellow); Retrosplenial-temporal (RSPT; white); Cingulo-parietal (cerulean); Low Signal (gray, outline in black). The Low Signal regions were excluded from analysis because they occur in known areas of signal dropout. Given their location (such as orbital frontal cortex and temporal pole) these regions have sometimes been called a cortical ‘limbic network’ in prior FC literature.

## Subcortical Signal Analysis

To assess the quality of BOLD signal in limbic regions, MR signal and standard deviation (SD - an indicator of noise) were interrogated. Fluctuations in BOLD signal contributes around 1-2% to SD. The remainder is accounted for by non-BOLD factors including respiration, pCO2 changes, vascular sources of noise, magnetic field changes, and movement [22]. Thus, when assessing signal-to-noise ratio, SD can be used as a proxy for noise. Raw magnetic resonance signal (‘MR Signal’ in Fig S1 & S7) was measured by taking the timecourse after preprocessing (realignment, distortion correction, resampling to 333 atlas space), and averaging across all timepoints that fell below the head motion threshold. SD was the standard deviation of the same timecourse.

The quality of limbic regions was assessed relative to extra-axial CSF (a negative control expected to have low BOLD signal and high noise-related signal variance), cortical parcels in areas of known signal dropout (expected to have low BOLD signal), and other cortical parcels (a positive control in that they should contain BOLD signal and relatively low noise-related signal variance).

## Serum Concentration Analysis

One subject had total ketamine serum concentrations that were consistently outliers, average 3.3 SD above the mean. When this outlier was removed, there was again no significant relationship between ketamine concentration and response to treatment. MADRS scores at enrollment and pre-treatment were not predictive of response at two or eight weeks (p>0.1).

|  | Pre-infusion | Day 1 | | Day 2 | Day 3 | Day 4 | Day 5 | |
| --- | --- | --- | --- | --- | --- | --- | --- | --- |
|  |  | AM | PM |  |  |  | AM | PM |
| Light-headedness | 5 | 9 | 5 | 70 | 76 | 48 | 33 | 5 |
| Headache | 14 | 0 | 0 | 17 | 5 | 5 | 5 | 0 |
| Palpitations | 5 | 0 | 0 | 0 | 0 | 0 | 5 | 5 |
| Disorientation | 0 | 0 | 0 | 4 | 14 | 14 | 0 | 0 |
| Restlessness | 5 | 9 | 0 | 9 | 14 | 14 | 10 | 5 |
| Nausea | 0 | 0 | 0 | 39 | 33 | 14 | 10 | 0 |
| Vomiting | 0 | 0 | 0 | 4 | 0 | 0 | 0 | 0 |
| Fatigue | 33 | 78 | 22 | 48 | 43 | 10 | 29 | 14 |
| Nystagmus | 0 | 0 | 0 | 22 | 29 | 14 | 0 | 0 |
| Pain | 25 | 4 | 9 | 13 | 10 | 0 | 0 | 0 |
| Inattention | 43 | 36 | 13 | 57 | 48 | 24 | 20 | 14 |
| Sedation | 0 | 35 | 22 | 43 | 48 | 33 | 14 | 14 |
| Diplopia | 0 | 0 | 0 | 22 | 38 | 19 | 24 | 0 |
| **# Responding** | **23** | **22** | **22** | **22** | **21** | **21** | **21** | **21** |

**Table S3. Side effects and adverse events (% of participants).**


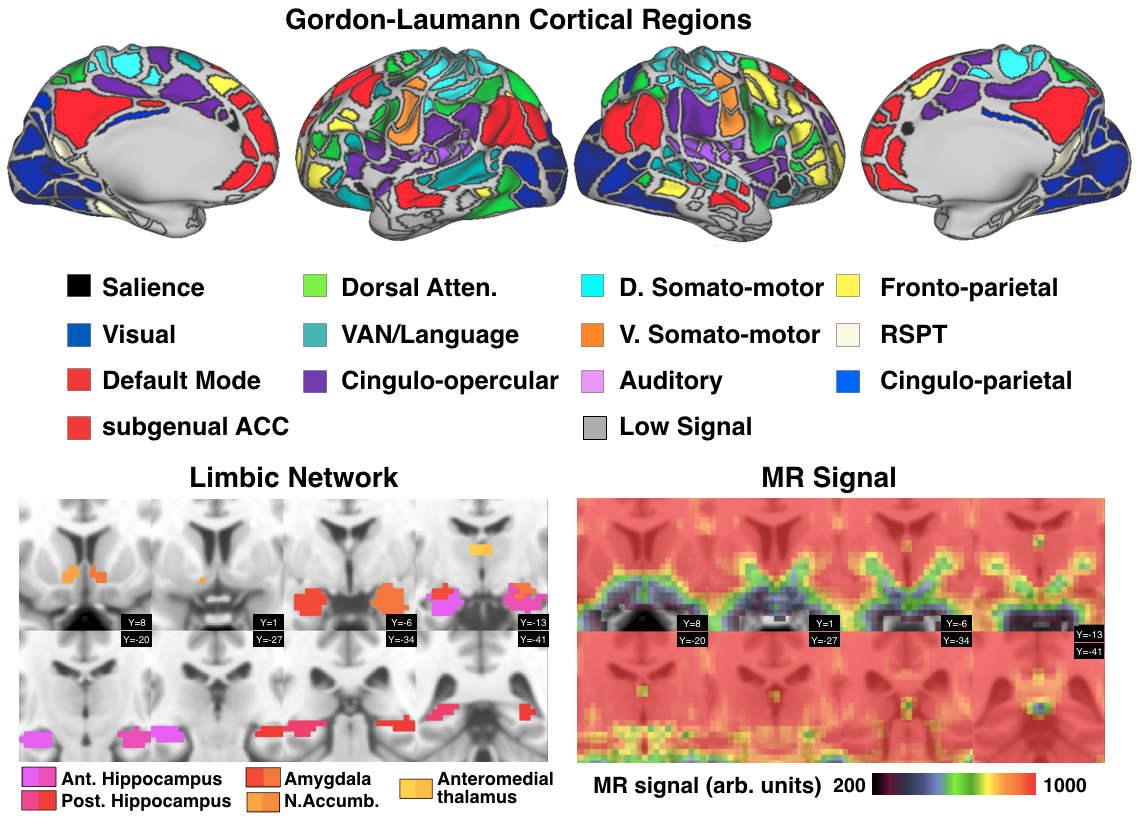


**Figure S1. Cortical and subcortical parcellation.** Top: Cortical parcels from the Gordon-Laumann parcellation, colored by resting state network membership. Bottom left: the ten regions included in the limbic network. Bilateral regions within the amygdala , anterior hippocampus proper, posterior hippocampus proper, and nucleus accumbens were individually defined based on freesurfer segmentation. Anteromedial thalamus was defined based on [23]. Legend indicates colors for left and right regions, respectively. Bottom right: A visualization of MR signal (generated by averaging functional volumes – see supplemental methods), demonstrates that some limbic regions (e.g. amygdala) overlap with areas of signal dropout, but also contain large areas of adequate signal coverage.


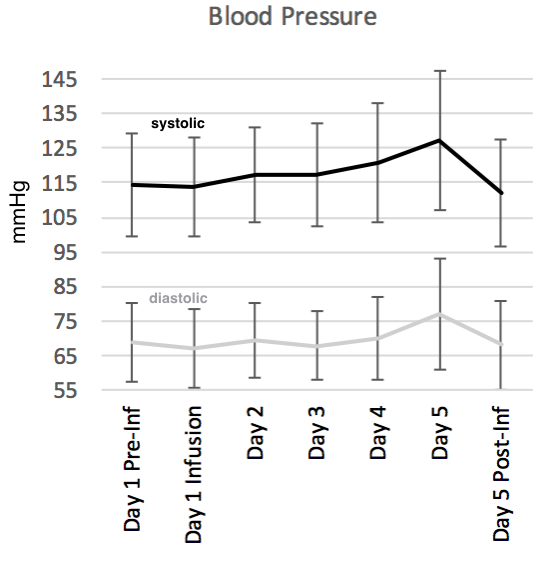


**Figure S2. Blood Pressure.** Average systolic and diastolic blood pressure (and SD) are shown across the 96-hour infusion and 1 hour after infusion was complete (Day 5 Post-Infusion).


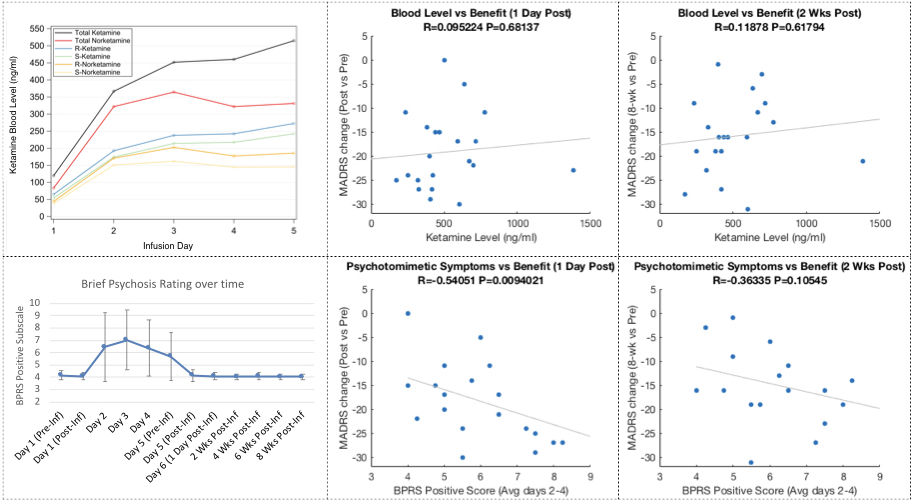


**Figure S3. Ketamine serum concentration (Blood Level) and BPRS.** Top left shows average concentrations of ketamine, its enantiomers, and its major metabolites. Top middle/right compare total ketamine concentration to MADRS change. Bottom left shows average Brief Psychosis Rating Scale positive symptoms score. Error bars indicate standard deviation. Bottom middle/right compare BPRS positive score to MADRS change.


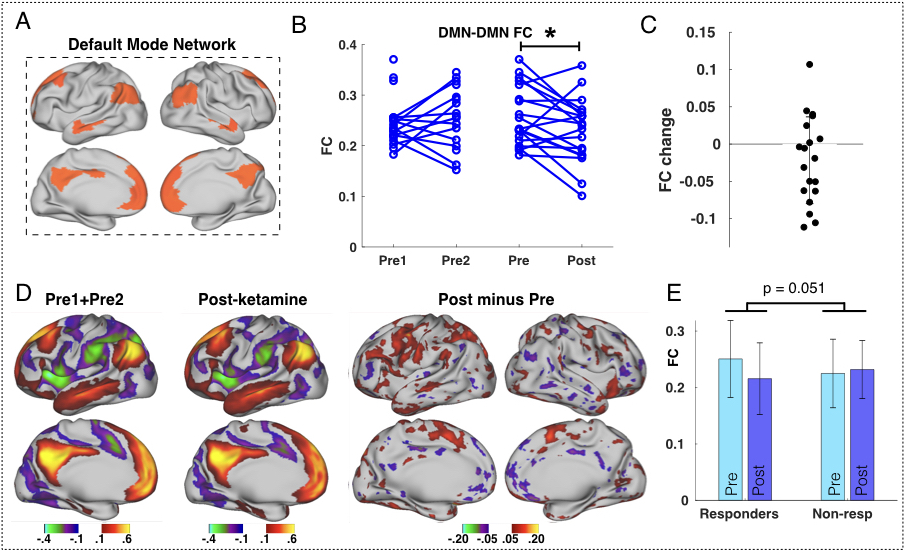


**Figure S4.** **Connectivity within the DMN decreases in responders.** A) Regions included in the default mode network. B) Blue lines on the left represent normal variability of FC in depressed patients (open circles are individuals with a single pre timepoint, black lines are mean of patients with both timepoints). Blue lines on the right represent change in FC resulting from ketamine infusion. Asterisk indicates that main effect of time (pre-infusion vs post-infusion) was significant (p = 0.003). C) FC change in TRD patients (post-infusion minus pre-infusion). Error bars = SD of FC change.. D) Maps of cortical FC of the DMN before and after ketamine. E) Comparison of FC change between responders (greater than 50% reduction in MADRS) and non-responders (less than 50% reduction in MADRS). Black bars on above indicate comparison of FC change in responders versus FC change in non-responders. Asterisk indicates the interaction of time-by-response yielded p < 0.05. Error bars = SD.


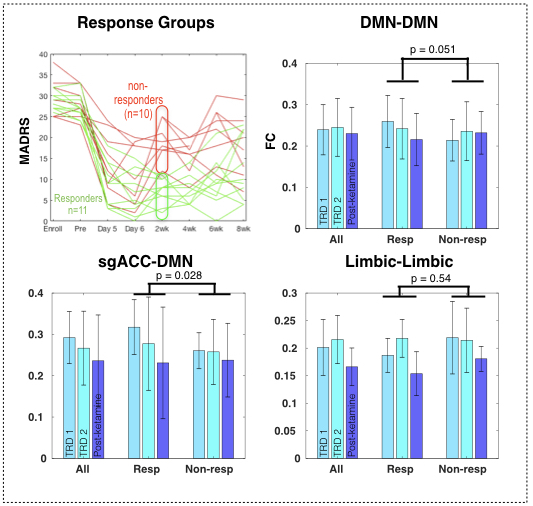


**Figure S5.** **Response Group Analysis.** Top left indicates response groups: responders (≥50% reduction in MADRS; green) and non-responders (<50% reduction in MADRS; red). For each of the three main FC comparisons, FC changes for all subjects, responders, and non-responders, are shown. P values indicate the significance of a time-by-response interaction in the mixed effects model. The statistical analysis was run after averaging pre-treatment timepoints, but here all timepoints are shown separately to enable visualization of pre-treatment FC variability. Error bars indicate group SD.


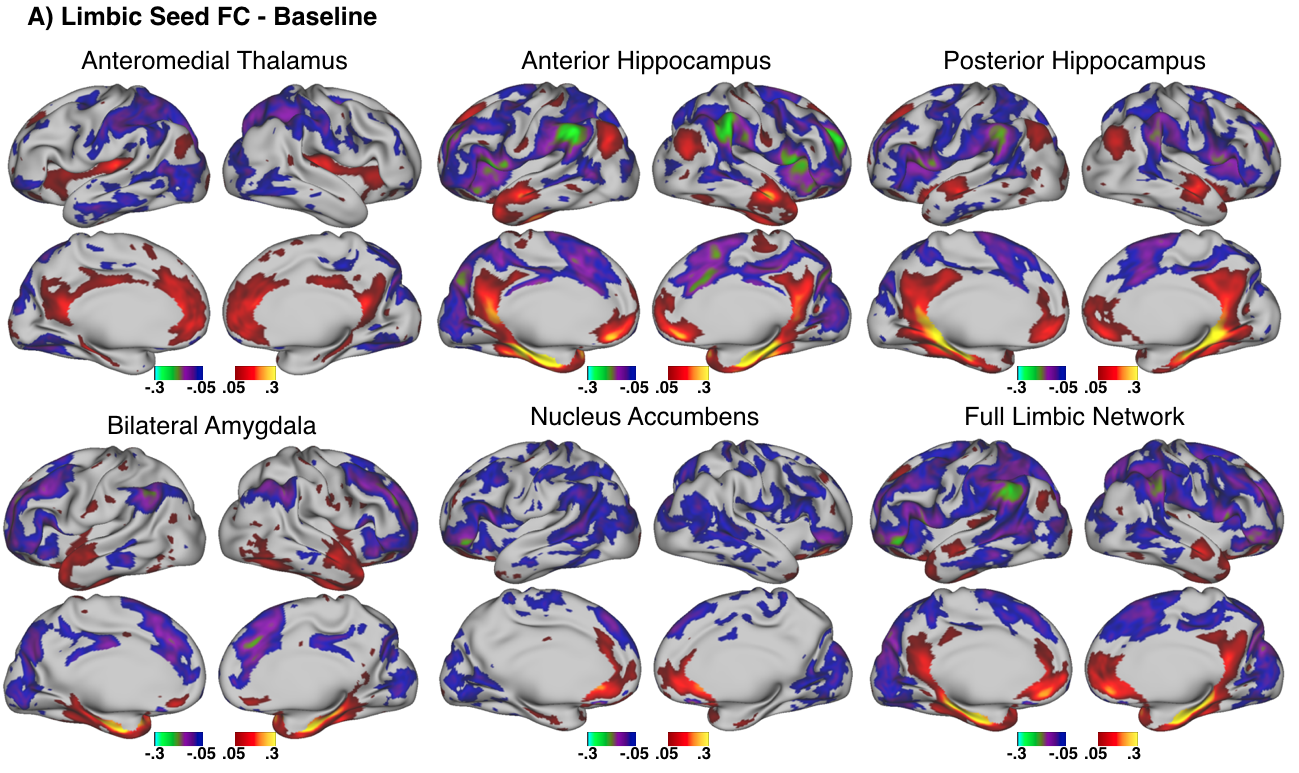

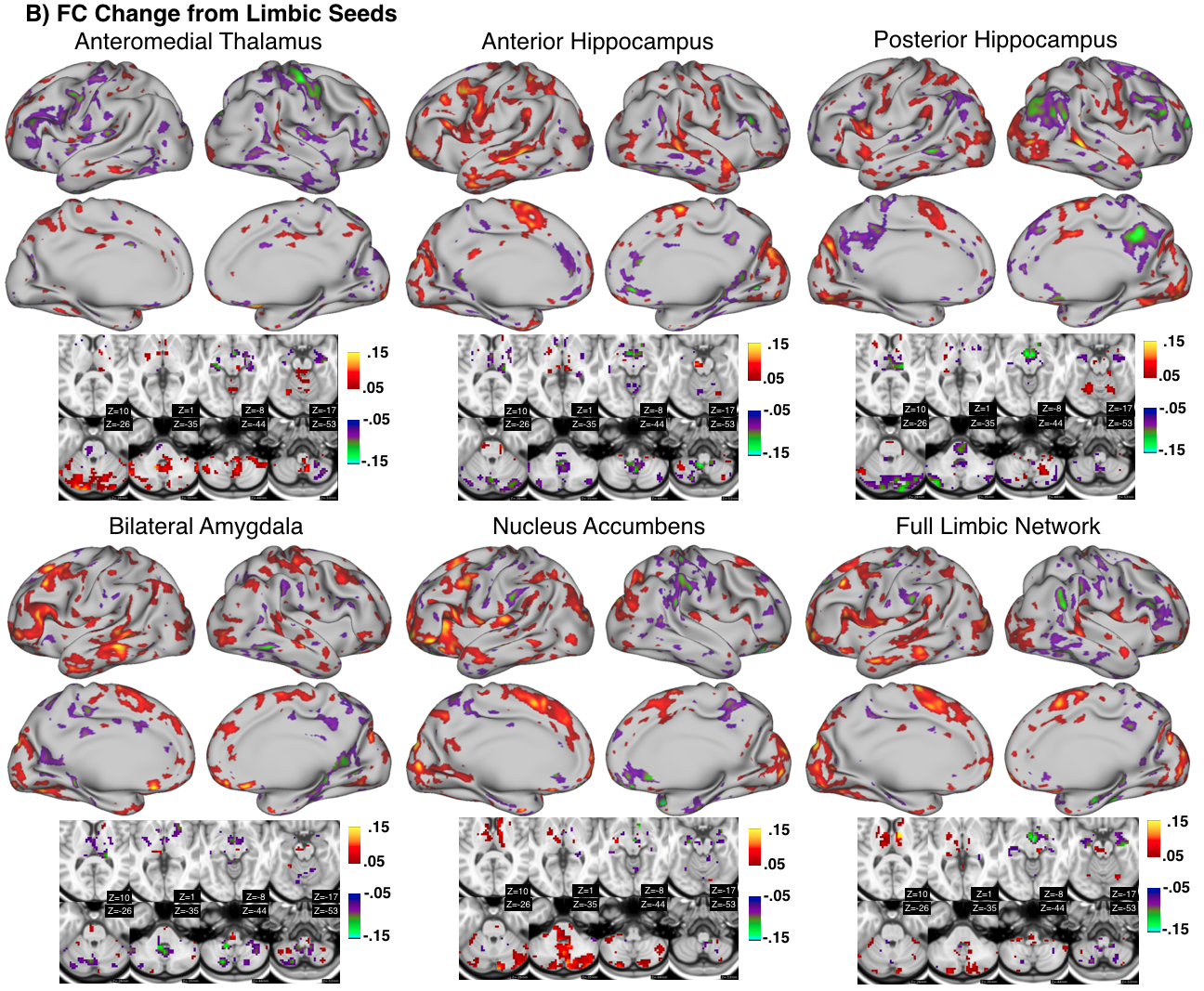


**Figure S6.** **FC from limbic seed regions.** Baseline FC (A) and FC change (B; post-ketamine minus pre-ketamine) from the each structure in the limbic network (using bilateral regions) and the network as a whole. In B, Red-yellow indicates FC increase following ketamine, blue-green indicates FC decrease.

**A**

**B**

**Figure S7.** **rsfMRI** **signal and noise in limbic regions. A)** Signal versus SD in different brain areas. Bold timecourse from timepoint 1 (after realignment, motion-scrubbing, and Goodvoxel mask; prior to nuisance regression and signal filtering) was taken from 391 regions: 280 cortical parcels (red), 44 cortical areas previously reported to have signal dropout (yellow), 35 limbic and subcortical regions (green, teal), 26 cerebellar regions (blue), 2 white matter regions (left and right hemisphere; purple), 4 CSF regions (ventricles & extra-axial; pink). Signal = the average BOLD value across all frames (raw MR signal); SD = standard deviation of BOLD signal across all frames (noise >> signal). Dots show values averaged across subjects. B) Signal versus SD at different stages of data processing. Pre-ketamine on the left and post-ketamine on the right. Top: same as A. Middle: removing high local variance voxels prior to averaging across voxels in a region both improves signal and decreases noise in may regions. Bottom: bandpass filtering and regressing nuisance timecourses (such as extra-axial CSF) reduces SD throughout, but disproportionately in limbic regions, such that they now have similar SD to cortex. For example, bilateral Amygdala (black ovals), has low signal and high noise (SD) relative to most cortical regions. By removing high-SD voxels and regressing various sources of noise (e.g. extra-axial CSF), SD is now similar to cortex. Note that because white matter and CSF regions are used to generate regressors, their SD drops to near zero after nuisance regression. Thus, they are excluded in the bottom panel.

## Supplemental References

1. Nurnberger JI, Blehar MC, Kaufmann CA, York-Cooler C, Simpson SG, Harkavy-Friedman J, et al. Diagnostic Interview for Genetic Studies: Rationale, Unique Features, and Training. Arch Gen Psychiatry. 1994;51:849–859.

2. Montgomery SA, Åsberg M. A New Depression Scale Designed to be Sensitive to Change. The British Journal of Psychiatry. 1979;134:382–389.

3. Petersen T, Papakostas GI, Posternak MA, Kant A, Guyker WM, Iosifescu DV, et al. Empirical Testing of Two Models for Staging Antidepressant Treatment Resistance. Journal of Clinical Psychopharmacology. 2005;25:336–341.

4. Farber NB. The NMDA Receptor Hypofunction Model of Psychosis. Annals of the New York Academy of Sciences. 2003;1003:119–130.

5. Sollazzi L, Modesti C, Vitale F, Sacco T, Ciocchetti P, Idra AS, et al. Preinductive use of clonidine and ketamine improves recovery and reduces postoperative pain after bariatric surgery. Surg Obes Relat Dis. 2009;5:67–71.

6. Trivedi S, Kumar R, Tripathi AK, Mehta RK. A Comparative Study of Dexmedetomidine and Midazolam in Reducing Delirium Caused by Ketamine. J Clin Diagn Res. 2016;10:UC01-04.

7. Lenze EJ, Farber NB, Kharasch E, Schweiger J, Yingling M, Olney J, et al. Ninety-six hour ketamine infusion with co-administered clonidine for treatment-resistant depression: A pilot randomised controlled trial. The World Journal of Biological Psychiatry. 2016;17:230–238.

8. Guy WE. ECDEU assessment manual for psychopharmacology-revised (DHEW Publ No ADM 76-338). Rockville, MD, US Department of Health, Education, and Welfare. Public Health Service, Alcohol, Drug Abuse, and Mental Health Administration, NIMH Psychopharmacology Research Branch, Division of Extramural Research Programs. 1976;1076:534–537.

9. Rowland DJ, Garbow JR, Laforest R, Snyder AZ. Registration of [18F]FDG microPET and small-animal MRI. Nuclear Medicine and Biology. 2005;32:567–572.

10. Fischl B, Sereno MI, Tootell RBH, Dale AM. High-resolution intersubject averaging and a coordinate system for the cortical surface. HUM BRAIN MAPP. 1999;8:272–284.

11. Siegel JS, Mitra A, Laumann TO, Seitzman BA, Raichle M, Corbetta M, et al. Data Quality Influences Observed Links Between Functional Connectivity and Behavior. Cereb Cortex. 2017;27:4492–4502.

12. Raut RV, Mitra A, Snyder AZ, Raichle ME. On time delay estimation and sampling error in resting-state fMRI. NeuroImage. 2019. 19 March 2019. https://doi.org/10.1016/j.neuroimage.2019.03.020.

13. Power JD, Mitra A, Laumann TO, Snyder AZ, Schlaggar BL, Petersen SE. Methods to detect, characterize, and remove motion artifact in resting state fMRI. NeuroImage. 2014;84:320–341.

14. Marcus DS, Harms MP, Snyder AZ, Jenkinson M, Wilson JA, Glasser MF, et al. Human Connectome Project informatics: Quality control, database services, and data visualization. NeuroImage. 2013;80:202–219.

15. Fox MD, Zhang D, Snyder AZ, Raichle ME. The Global Signal and Observed Anticorrelated Resting State Brain Networks. Journal of Neurophysiology. 2009;101:3270–3283.

16. Behzadi Y, Restom K, Liau J, Liu TT. A component based noise correction method (CompCor) for BOLD and perfusion based fMRI. NeuroImage. 2007;37:90–101.

17. Patriat R, Molloy EK, Birn RM. Using Edge Voxel Information to Improve Motion Regression for rs-fMRI Connectivity Studies. Brain Connectivity. 2015;5:582–595.

18. Glasser MF, Sotiropoulos SN, Wilson JA, Coalson TS, Fischl B, Andersson JL, et al. The minimal preprocessing pipelines for the Human Connectome Project. NeuroImage. 2013;80:105–124.

19. Satterthwaite TD, Elliott MA, Gerraty RT, Ruparel K, Loughead J, Calkins ME, et al. An improved framework for confound regression and filtering for control of motion artifact in the preprocessing of resting-state functional connectivity data. NeuroImage. 2013;64:240–256.

20. Yan C-G, Cheung B, Kelly C, Colcombe S, Craddock RC, Di Martino A, et al. A comprehensive assessment of regional variation in the impact of head micromovements on functional connectomics. NeuroImage. 2013;76:183–201.

21. Gordon EM, Laumann TO, Adeyemo B, Huckins JF, Kelley WM, Petersen SE. Generation and Evaluation of a Cortical Area Parcellation from Resting-State Correlations. Cereb Cortex. 2016;26:288–303.

22. Liu TT. Noise contributions to the fMRI signal: An overview. NeuroImage. 2016;143:141–151.

23. Seitzman BA, Gratton C, Marek S, Raut RV, Dosenbach NUF, Schlaggar BL, et al. A set of functionally-defined brain regions with improved representation of the subcortex and cerebellum. NeuroImage. 2020;206:116290.
